# Hospital Treatment for Wounded and Pensioned Soldiers

**Published:** 1917-10-27

**Authors:** 


### THE BRITISH HOSPITALS ASSOCIATION.

### HOSPITAL TREATMENT FOR WOUNDED AND PENSIONED SOLDIERS.

### A Call for Increased Payments.

### THE REMUNERATION OF ATTENDING PRACTITIONERS.

A well-attended Conference on these subjects was held in the Board Room of Westminster Hospital on Friday, the 19th inst., under the chairmanship of Mr. H. Wade Deacon, Chairman of the Liverpool Royal Infirmary. Amongst those present were: Rev. G. B. Cronshaw, Surgeon-General M. W. Russell, D.D.G.A.M.S., Sir Henry Burdett, Sir William Shipley, Mr. N. Bishop Harman, Mr. T. Hall Hall, Dr. James Neal, Mr. R. Ball Dodson, Mr. T. H. Lapthorn, Mr. J. W. Paton, Mr. H. W. Pollard, Mr. W. J. Locke, Rev. J. C. Pringle, Mr. H. R. Maynard, Mr. C. V. A. Horne, Mr. Thos. Hayes, Mr. G. Q. Roberts, Mr. E. W. Morris, Major W. McAdam Eccles, Mr. Edward J. Layton, Capt. H. S. Tunnard, Major T. Basil Rhodes, Mr. A. Leaney, Mr. J. W. Barnes, Mr. C. S. Risbee, Mr. L. G. Foster, Mr. Frank G. Hazell, Mr. W. J. Morton, Mr. Robert J. Bland, Mr. F. Neden, Mr. Herbert H. Jennings, Mr. Arthur Preston, Mr. A. Griffiths, Mr. Frank Inch, Mr. W. H. Harper, Mr. Gilbert Kempe, Mr. J. H. Shaw, Mr. Frank Oliver, Mr. H. W. Burleigh, Mr. J. W. Pearce, Mr. Henry V. Stuart, Miss A. E. Cummins, and the Honorary Secretaries, Messrs. J. Courtney Buchanan and Conrad W. Thies.

**The Chairman** explained that the Conference had been convened because several letters had been received from various hospitals in the country, drawing the attention of the Executive Committee of the Association to the trouble which has arisen concerning the cost of maintenance in hospitals; and, secondly, concerning the proposed payment for the hospital treatment of discharged and disabled sailors and soldiers. Clearly, if 28s. per week per patient was a proper figure three years ago, it could not be a fair figure for the same attention now, having regard to the greatly enhanced cost of living, and of drugs and appliances required for treatment.

**The Rev. G. B. Cronshaw,** (Treasurer of the Radcliffe Infirmary, Oxford), in opening the discussion, said that during the last four years some instructive figures had been supplied to him bearing on the first question to be discussed. These covered the four years to January last. The rate had risen in London hospitals, as much, in one case, as from 3s. to over 14s. per day per patient. In London the costs per patient were thoroughly investigated by the War Office, but he felt justified in saying that investigation was much to the benefit of the War Office. He did not think those who conducted it really understood the case. The importance of the matter was felt so keenly by hospital managers that they thought the Association should come to their relief. The hospital wards now contained less men patients than in normal times, owing to the shifting of the civil population into the Army. Therefore any contribution was of value, and the hospitals were now receiving something they could never get if there were not this contribution from the War Office. On that basis, he supposed there were some people who would be willing to accept 1s. per bed per day. There was a basis which members of the Association felt was the right one—namely, that the contribution should approximate to the cost of the hospital, and that the management should not be required to call on the subscribers' money, or upon the hospital capital, too largely in order to pay for what the Government was responsible for. And what made many responsible people in hospitals anxious to have the matter discussed was that there was a sort of wide maximum in the War Office figures which local administrators were allowed to play with, and some of the smaller hospitals had been raised to the larger figure, thus making their costs become equivalent to those of the bigger hospitals, and more than 4s. per day could not be drawn. The larger hospitals wanted more because the smaller hospitals had had their quota added—and they thoroughly deserved it—and the larger institutions felt they deserved it as well. On that basis most of the members wished to see what could be done with the War Office by standing together, as would be implied by passing the resolution he intended to move, and asking that the upper figure might be raised so as to be more in accordance with present prices. The case would need to be carefully prepared and the costs taken out in detail. The War Office did not seem to understand how hospital costs were made up. On the figures which Mr. Hayes had supplied to the hospital committee, a very good case could be made out covering the last three years, the figures all having been duly audited. They did not include the heavy costs which had necessarily been incurred since January 1917. On behalf of the Executive, he had much pleasure in moving :—

" That, having regard to the abnormal advance in prices of provisions and all other commodities since the rates of payment for the treatment of wounded soldiers in civilian hospitals were fixed, this meeting is unanimously of opinion that formal representations should be made to the authorities concerned, with a view to obtaining an immediate increase in such rates of payment."

The resolution having been seconded,

**Mr. Lapthorn** (Portsmouth) said the resolution assumed that the question for the meeting to discuss was purely one as to whether the proposed contribution by the War Office was sufficient. But he had hoped that the larger question would have been discussed to-day—namely, the relation of the hospitals to the War Office in view of future as well as existing conditions. The offer of the War Office was to pay for maintenance only, and he did not think the offer went far enough, for the Department should provide accommodation also. He hoped the meeting would discuss the latter point. He knew some hospitals were in the happy position of having ample accommodation for their civilian requirements, and a little to spare; but that was not the case with the hospital he represented, for before the war it was all they could do to meet the civilian needs. They were also without the space which would enable them to meet the enlarged demands which would probably arise from the admission of injured sailors and soldiers after the war. Perhaps he would find himself in a decided minority, but he

**BRITISH HOSPITALS ASSOCIATION**—(*continued*).

wished to submit to the meeting the principle that the full responsibility of providing not only maintenance, but accommodation, for all sailors and soldiers who were injured or impaired in health through war conditions was a debt which the State owed to those men, and which it had no right to expect other people to pay. If that principle were admitted, it followed that a grant ought to be asked for not only maintenance, but for building purposes, in the form either of rent or a capital grant to enable those who needed to do so to enlarge their accommodation. He realised this would bring the objection that it meant State help, and carried with it, of necessity, some form of State control. But there was no different principle involved in that than in receiving payment for maintenance; and even if some form of State control were involved, he believed it would be possible to combine that with all the best features of voluntaryism, particularly with the retention of the voluntary spirit of boards of management. He had no desire to side-track the resolution before the meeting, but he would like to move that, while the hospitals were willing to place all their resources at the service of the injured men, they considered that the full responsibility, not only of maintenance but also of providing accommodation, should be accepted by the State.

**The Chairman** preferred not to accept such a resolution, as it opened up a much larger question.

### The Needs of the Population.

**Mr. N. Bishop Harman, F.R.C.S.** (West London Hospital) said that if the hospitals were paid to the full for the expenditure incurred by them for the treatment of sick and wounded soldiers there would be every inducement for them to extend the amount of accommodation which they were already providing for such soldiers, to the detriment of the claims of the civilian poor, for whose treatment voluntary hospitals were established. At the beginning of the war there was a great manifestation of patriotism; there was every wish to help the Government by throwing the civilian hospitals open to sailors and soldiers. But it was only meant as a temporary measure. Civilian patients were now largely the young and those too old for military service, and they were to a considerable extent pressed out of their rights to proper surgical and medical treatment. He therefore regarded this resolution as unwise. The question came up before a representative meeting of the British Medical Association, and a resolution was passed—he believed unanimously—recommending that the attention of the authorities be called to this, and that the accommodation which had been provided for the sick poor should be retained for them. At the West London Hospital three wards had been given up to soldiers. As this had resulted in an enormous "waiting-list" for civilian patients, a protest was lodged, with the result that two had now reverted to civilian patients. The hardship due to the reversion was not great on the soldiers, because the latter were mostly convalescent, and not of the severity of disease for which ordinary patients were admitted to the beds. A lady recently told him that hospital boards were doing great detriment to the cause of the hospitals by taking in so many soldiers, for whom the State was bound to provide; and that next time support was asked for extra accommodation she would ask what was done with the beds during the war. There was, Mr. Harman continued, plenty of room for the treatment of sick and wounded soldiers without trenching on the claims of the sick poor. He proposed a resolution embodying his views, but it received no seconder.

**Sir Henry Burdett, K.C.B.**, answering the last speaker, said everybody present who had to do with hospitals, and all residents in the British Isles, desired that the civilian population should have adequate hospital treatment. But we were now in a state of war, and on the result of that war depended the very existence of our nation. It was not a question only of the civil population, but of the whole population of these islands, their medical care, their welfare, and their very existence. The system we were fighting against was not one that we Britons could live under, and the way to provide adequately and speedily for the civilian population was to get on with the war. Having won the war, this and other matters could be put right. In the meantime it was up against every governing body of a voluntary hospital to do their utmost to provide for all civilian cases which urgently needed hospital care. So far as his information and observation went—and it had been extended and continuous throughout the whole period of the war—there was not a single well-administered hospital which had not enforced that policy to the full. Mr. Harman had only touched the fringe of the subject. He (Sir Henry) had felt keenly the injustice to the members of the medical profession which they had suffered in war-time, but he thought that at the moment all they could do was to try to make the best of the conditions of war by providing for our fighting men first, and also adequately for the civilians and practitioners concerned.

**Mr. Stuart** (North Staffordshire Infirmary) asked whether, in presenting the case, the War Office would be asked to take into consideration administration as well as maintenance.

**The Chairman** said he could not answer for the Council, but the resolution had been drawn on broad lines, and whatever the 28s. weekly per bed included it was not adequate at present.

**Mr. Stuart** said his committee would like out-of-pocket expenses to be included, but they did not want to feel that the hospitals were making money out of this arrangement.

**Mr. Dodson** (Royal Sussex County Hospital) pointed out that the cost of administration was great in the case of soldiers, because of the number of forms to be filled up—in many cases eight times over for one man. Mr. Harman had lost sight of the fact that practically all men between twenty and forty years of age were now treated as soldiers, whereas formerly they were civilian patients. Among women patients there had been a definite diminution, probably because those who formerly came with nerve troubles were now too interested and occupied to be ill, and also because so many of the derelicts of the population were now earning good money. In his own hospital sixty-five extra beds had been put in. Up to a certain point the hospitals could be placed at the disposal of the War Office, but there came a stage at which it was necessary to think carefully of the money due for the treatment of the civilian poor. At the present time that point had been passed. He felt that so long as the cost was not more than 1s. extra—*i.e.*, so long as it was under 5s.—no complaint would be made. But the requirements of the War Office in filling up forms, etc., greatly increased the administrative costs. More was also needed for the payment of medical men and nurses. Before the war his hospital was under contract with the War Office for military cases, and 6s. per day per patient was paid. At the outbreak of the war his hospital offered the War Office 100 beds at the price they had been paying,

**BRITISH HOSPITALS ASSOCIATION**—(*continued*).

and it was gratefully accepted. Two months later they were asked to accept 4s. in lieu of 6s., and agreed to do so under the circumstances. At that time the daily cost per bed was 4s. 1d. In 1917 it was probably over 6s., in which case one-third of the cost of military cases was coming out of charitable funds. An appeal he made to friends in America for help was declined, since the English War Office paid only two-thirds of the cost of their patients.

**Major McAdam Eccles, M.S.** (St. Bartholomew's Hospital) said the first question was as to whether soldiers, disabled by wounds or sickness, should be treated in civilian hospitals at the expense of their charitable funds. If so, there remained nothing further to be said. If, on the other hand, it was agreed that it was not right—for it was being done at the present moment—it was for hospital administrators to see that the payment received covered the whole of the cost. The cost included three things: (*a*) cost of maintenance of the wounded or sick soldier in the civilian hospital; (*b*) the increased cost of administration, which was considerable in the case of the soldier; (*c*) the charge for medical services, which would have to be an increasing figure. It was not right to have to treat State patients without payment. That would be an important consideration in regard to the second resolution to be discussed, for the wounded soldier would be with us for many years after the war. The payment for medical services should not be made direct to the medical staffs but to the lay administration, and the latter should hand over what they considered to be the right proportion over and above the cost of maintenance and administration to the medical staffs as a collective body.

**Mr. Griffiths** (Ipswich) said he came with the authority of his board to support any resolution of the Association for an increased grant. They had been pioneers in this matter, for they obtained the maximum of 4s. in September 1914. His hospital had 150 beds for civilian patients, and had put up accommodation for 280 wounded. They had practically no waiting-list for civilian patients. The extensions had been paid for by local subscriptions, to the extent of £10,000. His board thought it undesirable, at this juncture, to ask for a grant-in-aid from the War Office. The matter was two-fold: they wanted to help the War Office and they wanted to help the country. They asked the War Office what it cost to treat patients in military hospitals, and the reply was to ask his board to confer with them with a view to a possible reduction in the cost of maintenance. (Laughter.) A further inquiry to the same effect brought no response. He cordially supported the resolution as submitted.

**Mr. Hazell** (Manchester) thought one effect of the discussion should be to make hospitals practise more rigid economy, especially in the matter of deciding on the best dietaries for patients on a scientific basis—*i.e.*, according to the calories required. This exactitude should be extended also to the articles used for laundry and other purposes, the purchases being determined upon results of analyses by the hospital pharmacist. There never ought to be such a variation in the same city as from 3s. to 14s., as the opener mentioned. His experience showed that 4s. 6d. was a proper figure, though it would vary a little with the particular town. Patriotism entered largely into the matter at the commencement of the war. Some hospitals received 2s., some received nothing, some had 4s.

The resolution was carried, with one dissentient vote.

### Payment for Professional Services.

**The Rev. G. B. Cronshaw** proposed: "That this meeting is of opinion that the scale of charges for the treatment of disabled sailors and soldiers in civilian voluntary hospitals as proposed by the Ministry of Pensions (*vide* Schedule I., 'Instructions and Notes on the Treatment and Training of Disabled Men') is inadequate, and would only partially meet the cost of such treatment. The meeting further strongly affirms the view that the scale of charges should be such as would cover the whole cost of the maintenance, including treatment given, and remuneration in respect of professional medical services." He said this laid open an entirely new principle, and concerned the carrying on of abnormal work for many years to come. No one could say that hospital managers had been profiteering. The National Insurance Act brought much to their doors, and but for the work which the hospitals of the country had rendered to the National Health Commissioners that Act would have aborted long since. The same could be said in regard to the campaigns against tuberculosis and venereal disease, despite the depletion of hospital staffs; they had stood as trustees to the nation and had done their duty. Probably in the future they would be required to take an even larger share. He had stood for voluntaryism as long as he had known anything about hospitals, and he hoped to do so throughout his life. It must be remembered that if the voluntary hospitals did not accumulate round themselves the work which was being given them to do, it would go elsewhere; the Ministry of Pensions would set up its own hospitals in various parts of the country, take their patients away, and put them into their own hospitals at their own cost. No one would allow a repetition of the sequel of the South African War, when soldiers injured therein were begging to the public for charity. On the walls of this hospital in which they were meeting was a notice to the effect that no soldier was to receive charity; he was to have things as a right. The Pensions Ministry did not want to set up special hospitals, the cost per head would be too great, so they came to the voluntary hospitals to do the work. He was willing to shoulder that work. The hospitals were now in a difficult time, not only because of the war, but because much the same obtained previous to the war. Some hospitals found their subscriptions were almost at the vanishing-point, and their legacies got smaller year by year. On the other hand, there was *x*-ray work and other special treatment which increased in cost almost daily. The money involved came to thousands, and the new situation had to be faced. And most hospitals desired to increase their maternity work so as to do their best to conserve infant life. The second question concerned payment, and there could be no doubt that the Government ought to provide for the complete maintenance and administration of these beds which they might take for disabled sailors and soldiers. With regard to payment of the medical staff, he thought the proper way was for the hospital to take the payment and contribute a fixed sum to their staff through a fund, leaving the staff to settle how they would apportion it among themselves. The questions to be decided were: (1) Were they prepared to do this, recognising the changing conditions? (2) What sum should be accepted for it? And it should not be simply the sum which the Pensions Ministry might decide would suffice, but the hospitals should be taken into consultation in the matter, and show what the costs were. Despite the requirements of the King's Fund and Sir Henry Burdett, it was difficult to separate out-patient costs from in-patient costs. But

**BRITISH HOSPITALS ASSOCIATION**—(*continued*).

hospital managers knew what *x*-ray work, massage, and other special treatments cost, and the figure would perhaps astonish the Ministry of Pensions! One had only to look through the figures of the King's Fund to see how the charge came out per out-patient and per bed, according to the needs at the several hospitals. And it must be remembered that hospitals were dealing with markets which were easy at one time and hard at another.

**Mr. Roberts** (St Thomas' Hospital, London) seconded the resolution. It must be remembered, he said, that these sailors and soldiers were suffering and had been discharged from the Services as the result of their activities in the war, and his hospital had already had a wide experience of the kinds of cases which this resolution referred to. They were certainly very difficult cases to deal with, and fully-equipped hospitals should be entirely at their service, especially as the treatments needed embraced a wide variety. But this work could not be done at the tentative rates given by the Pensions Ministry without incurring loss. The first proposal, he understood, was that general out-patients would pay 1s. for the first attendance and 6d. for each subsequent attendance, the charge being raised to 2s. 6d. the first time and 1s. 6d. each subsequent visit for treatments which could be regarded as special. These two categories caused a complication, and he much preferred a flat rate for all, especially as it would be impossible to say at first what the rate should be in many cases. Anything less than 2s. per visit would not pay the hospitals, especially as there would be much detail as to diagnosis, history, previous modes of treatment, and results to be mastered, and there would also be considerable correspondence. The staff had the right to ask why, when the State was prepared to pay for the treatment of these men, they should be required to do their important work for nothing. It was essential that some scale of payment should be fixed for the medical staff. Some representatives of the Association ought to consider the sum which should be asked so as to go to the Ministry of Pensions with a definite proposal. He did not doubt that the Ministry would meet them fairly in the matter; 4s. a day did not pay for in-patients anywhere; he agreed it was nearer 6s. He believed the staff would rather retain their present position as voluntary officers visiting in the wards and not expect payment in respect of their in-patients.

**Sir Henry Burdett** said there was no treasurer who had worked more actively for, or rendered better services to, voluntary hospitals than Mr. Cronshaw. He had devoted his energies continuously to the study of hospital administration in all its branches, and covered the whole field. Indeed, his was a splendid example of what a capable treasurer could accomplish for an important hospital like the Radcliffe Infirmary, Oxford. The Association must feel grateful to Mr. Cronshaw for bringing two important questions before the members and for the inspiriting example he had set. Sir Henry hoped this resolution would be carried unanimously, and for three reasons. First, the whole nation was unanimous that every sailor and every soldier who fell out by the way during the present war must be provided for, not by charity, nor by the Poor Law, but by the State. It was his privilege to-day to come to that meeting direct from the Front, where he had spent some weeks in the hospitals, camps, and with the various Services and workers, from the advanced posts through the clearing stations and hospital trains to the base hospitals. He also had the privilege of being under fire when in the open for 1½ mile. His first message from the Front was that there was an atmosphere about the work which he heartily wished could exist and be realised by everyone at Home. Men—combatants and non-combatants—and women were working hard and continuously, without relaxation and under almost continuous pressure. It was not unusual for a hospital staff to have to be operating continuously from 9.30 A.M. until midnight, keeping that up for seven or eight days. Sometimes hundreds of cases had to be evacuated for new patients from the Front, and that had to be done between midnight and 6 A.M. This would give some idea of the devotion of the workers. The message he brought back was that there could be nothing too good for the fighting men. That he regarded as an unanswerable justification for this resolution. It was thought by many that the step proposed to be taken might injure the voluntary system. But the war had taught the necessity for holding large views and looking ahead. The war would entail, among other things, a thorough reorganisation of our hospital system. All over the country were a large number of new buildings devoted to the care of patients, and those buildings would to a large extent be evacuated after the war. It was for the hospital managers in every part of the country to make themselves familiar with everything of the kind which existed around them, because it was believed that the best of them might be brought under their management ultimately. With regard to the medical staffs, the doctors who were working for the hospitals had rights which needed to be considered. Many of them had given up all their time and had gone on steadily increasing the number of their services so as to provide medical care for the new departments to which Mr. Cronshaw referred! In arriving at this resolution the Association had for the first time created a basis of justice for the medical profession which their services throughout the war abundantly justified.

**Mr. Burleigh** thought the voluntary hospitals might now be standing at the parting of the ways, and that if they refused now to accept the responsibilities which the War Office, the Ministry of Pensions, and the London County Council asked them to accept, the ultimate state of those hospitals might be serious. Honorary physicians and surgeons would not expect payment, but they suggested that out of the capitation grant resident physicians should receive a salary, so as to attract to the service of the institution good men who otherwise would enter general practice. He agreed that a flat rate would be best.

**Mr. Barnes** (Sheffield Royal Infirmary) said the two local hospitals in Sheffield passed a resolution unanimously that any payments made to voluntary hospitals for the treatment of men for whom the Ministry was responsible should include an amount earmarked for the medical staff, and entirely at their disposal. He had been authorised to ask for the views of the meeting on this point.

**Major McAdam Eccles** suggested one or two amendments in the wording of the resolution, which would cause it to read: "That this meeting is of opinion that the scale of charges for the treatment of disabled sailors and soldiers in civilian voluntary hospitals, as proposed by the Ministry of Pensions (*vide* Schedule I., 'Instructions and Notes on the Treatment and Training of Disabled Men') is inadequate, and would only partially meet the cost of such treatment; and strongly affirms the view that the scale of charges should be such as

**BRITISH HOSPITALS ASSOCIATION.**—(*continued*).

would cover the whole cost of the maintenance, including treatment given and including some remuneration in respect of professional medical services." Those who had been working for three years or more in our large military hospitals felt strongly that those men who had been wounded and disabled for our sakes ought to have a very great hold upon the whole of the population, and that in the future these men were not to be treated by charity. There were two ways in which they could be treated by charity: (1) By the charitable public, and to a large extent they were being so treated by them; (2) by what was called the "charity" of the poorhouse. They were determined, so far as London was concerned—and the provinces were following too—that the civilian voluntary hospitals should treat these men, but that the cost of the treatment should not fall on the charitable public. It was the duty of the Government, through the Ministry of Pensions, to see not only that these men had their pensions, but that they were brought back again in economic value to the nation at large.

A member asked whether the Executive Committee would consider it an instruction to ask for a payment for the use of buildings.

**The Chairman** replied that he would propose that the matter be referred to the Council. The Executive Committee was a small body, whereas the Council consisted of fifteen to twenty representative members.

Government Departmental View.

**Mr. Horne** (representing the Ministry of Pensions) said efforts had been made by his Department to ascertain what were the charges for in-patient and out-patient treatment. Conditions had changed rapidly in the last six months. It was important to arrive at a uniform charge, and the bodies which had been consulted told them the figure mentioned might be regarded as a uniform charge. But the instructions showed that the Ministry had left room for any case of a hospital which could show that it gave a special type of treatment, such as that for paraplegics; and in those cases the Ministry had not hesitated to sanction a higher charge. Several speakers had pointed out that the hospitals were coming in for a much bigger share of public work, and he was glad of that, because he did not think anyone could do it better. The Ministry had no intention of setting up in various parts of the country institutions which would compete with the voluntary hospitals. They had, in certain instances, provided institutions of which there was a dearth, such as those for neurasthenia, tuberculosis, and epilepsy, particularly the last-named. Generally, the accommodation was provided by enlarging existing institutions, and in time those institutions would derive the sole benefit from the extra accommodation. The basis of the charge taken was the amount to which the hospital was out of pocket by the patient. There was no intention that the institutions should be the loser by such patients: the Department was not out to drive a keen bargain. The question as to whether every single item could be brought up in the accounts as a business concern, including perhaps remuneration for the doctors, was a larger matter, one which must be settled as a general policy of the State. He thought it would be very difficult to get through the Houses a Ministry of Health Bill during the next three years. Even if such a Bill remotely approached that which was attempted in the last year, it could not avoid the question of hospital treatment, which was deliberately left out of the Insurance Act. And local authorities had not been encouraged by the Local Government Board in past years to put up hospitals; that had been left to voluntary effort. But no Ministry of Health could avoid considering the hospital question as a whole. As soon as one got State maintenance in these hospitals, it would raise the question of the terms on which payments could be made, and, as a corollary, the conditions which the hospitals would have to satisfy, if they were to charge everything, not only rates, rent, taxes, and insurances, but also the medical services of the staff. With regard to the particular action of the Ministry of Pensions, the matter was looked at in the following way. The problem of treatment of the discharged man was clearly a temporary matter, in the sense that if one took 50 per cent. of the pensionable cases with wounds and visible injury within six months after the man's discharge—in many cases within three months—the man was as far healed as he would ever be after discharge from the military hospital. Or taking such complaints as neurasthenia and others for which discharge was given, within possibly a year of discharge the man was no longer requiring hospital treatment or specialist treatment. If that were so, assuming the war were over next year, in three years' time the problem would have ceased to be a problem at all. Therefore, for the Ministry to have gone into the question of whether the principle on which the charge that hospitals were making to existing bodies was the proper one, or whether they should entirely revise that scale and introduce a new principle—on which doctors said there was considerable difference of opinion—would have prejudiced the other bodies who were dealing with the hospitals, would have involved long negotiation with those other departments and bodies, and would have delayed seriously the work which they had in hand. Those instructions were very carefully thought out over a period of six months, and they were based very largely on the experience of other departments. And their feeling was that, while in no case did the Ministry want a hospital to be at any loss by that uniform charge when it was giving treatment of a more expensive kind than usual, it was difficult for the Ministry of Pensions to go into a general question, though it might have its merits for a Ministry of Health. The Ministry of Pensions had given an assurance that no terms which were accepted by hospitals now under these instructions would prejudice its view of a general settlement on whatever lines the profession and the public, or whatever other departments would have charge of it, might arrive at as a proper solution of the problem.

**The Chairman** thought it was evident that the meeting had come to the conclusion that some payment should be made for services rendered; and it seemed to him that if medical men had decided that they must be paid some arrangement would have to be made.

The resolution was carried unanimously.

**The Chairman** proposed: "That the Chairman and Council of the British Hospitals Association are hereby requested to consider the foregoing resolutions and to take such action as may be necessary." Carried.

**The Chairman** intimated that Lord Sandhurst and Sir Arthur Stanley had sent messages regretting their inability to be present, and offering their assistance should a deputation be sent to the authorities. Answering a suggestion, he promised that the hospitals should be informed as quickly as possible.

Votes of thanks to the Chairman and to the Westminster Hospital authorities for the loan of the room terminated the proceedings.

